# Perspectives of Young Adults on Receiving Telepsychiatry Services in an Urban Early Intervention Program for First-Episode Psychosis: A Cross-Sectional, Descriptive Survey Study

**DOI:** 10.3389/fpsyt.2020.00117

**Published:** 2020-03-03

**Authors:** Shalini Lal, Amal Abdel-Baki, Sunil Sujanani, Florence Bourbeau, Imaine Sahed, Jessica Whitehead

**Affiliations:** ^1^ School of Rehabilitation, University of Montreal, Montreal, QC, Canada; ^2^ Youth Mental Health and Technology Lab, University of Montreal Hospital Research Centre, Montreal, QC, Canada; ^3^ PEPP Montreal and ACCESS Open Minds, Douglas Mental Health University Institute, Montreal, QC, Canada; ^4^ Department of Psychiatry, Centre Hospitalier Université de Montréal (CHUM), Montreal, QC, Canada; ^5^ Axe Neurosciences, University of Montreal Hospital Research Centre (CRCHUM), Montreal, QC, Canada; ^6^ Department of Psychiatry, Université de Montréal, Montreal, QC, Canada

**Keywords:** information and communication technologies, mhealth, e-mental health, mental health services, psychiatry, telemedicine, homeless, video

## Abstract

**Background:**

Limited knowledge exists on telepsychiatry in specialized services for first-episode psychosis (FEP), despite its potential for improving service access and engagement.

**Objective:**

To explore access and use of technology, obstacles to attending clinic appointments, and perspectives of young adults with FEP on using telepsychiatry as part of outpatient services.

**Methods:**

A cross-sectional, descriptive survey study was conducted between July and October 2017 with young adults between the ages of 18 and 38 recruited from a specialized program for FEP in an urban Canadian setting. Data were analysed using descriptive statistics and content analysis.

**Results:**

Among 51 participants (mean age = 26.1, SD = 4.2; 59% male; 20% experiencing housing instability), more than half (59%, n = 30) rarely or never used mainstream video chat (e.g., Facetime). The majority (78%, n = 40) reported obstacles to attending appointments, with several (37%, n = 19) identifying two or more. Almost half (49%, n = 25) were very favorable towards telepsychiatry and a quarter (25%, n = 13) were somewhat favorable. Participants expressed several concerns about telepsychiatry, including loss of human contact and confidentiality.

**Conclusions:**

To our knowledge, this is a first study on the perspectives of individuals with FEP about telepsychiatry. Despite experiencing obstacles to attending appointments and expressing receptivity towards telepsychiatry, participants did not have access to these services. It is important to provide education to clinicians on the potential of telepsychiatry to improve service access. Also, more research is needed on when, where, and how telepsychiatry can be integrated into existing care while addressing patient and clinician concerns.

## Introduction 

Young adults with first-episode psychosis (FEP) face individual, cultural, service, and system-level obstacles to engaging with mental health care (1). It can take half a day, or even more to attend a follow-up appointment (for example, after being discharged from the hospital, or after receiving an initial psychiatric evaluation as an outpatient), including time for transportation, waiting to be seen, and coping with other factors. Moreover, this process may need to be repeated on a weekly or bi-weekly basis at different time points during one’s follow-up, which may not always be feasible due to competing priorities (e.g., caregiving, school/work schedules, financial resources for transportation).

To address these challenges, many early intervention programs for psychosis have adopted a community-based approach whereby clinicians deliver services outside clinic walls (2). However, this approach requires time for travel and the young person may not always be present or receptive to community visits. New models that are efficient and effective in sustaining engagement of young people are needed. Leveraging technology-supported services may help with service engagement (1, 3) and preliminary studies (4–7) conducted with the FEP population indicate that they have access to and use mainstream technologies (e.g., Internet, computers, mobile phones); however, limited attention has been given to the perceptions of young adults with FEP on use of telepsychiatry to receive mental health services.

Telepsychiatry (or tele-mental health) involves real-time communication with a mental health care professional through secure video conferencing solutions. Systematic reviews conducted with the general psychiatric population have shown that telepsychiatry is reliable for conducting assessments, equivalent in terms of producing treatment outcomes, and is cost-effective in comparison to face-to-face sessions [e.g., (8)]. However, as illustrated through Shore’s review (9), most telepsychiatry studies have focused on rural and remote settings. Research is needed to better understand if individuals living in urban contexts face barriers in accessing services that could be addressed through telepsychiatry.

Moreover, very few studies have investigated the use of telepsychiatry with individuals diagnosed with FEP or schizophrenia-spectrum disorder. For example, Kaskow et al.’s (10) telepsychiatry review found only 6 studies conducted with participants diagnosed with schizophrenia and more recently, Santesteban-Echarri et al. (11) found 14 studies, though none were clearly focused on a population with FEP. Nonetheless, the results of these reviews provide preliminary evidence that telepsychiatry services for patients with schizophrenia-spectrum disorder is feasible and acceptable. However, limited, if any, research exists on telepsychiatry in urban and rural early intervention programs for psychosis, from the perspectives of patients, support networks, and service providers.

The purpose of this study was to better understand the perspectives of urban young adults with FEP on receiving telepsychiatry services as part of their follow-up (e.g., after being discharged from the hospital or following an initial outpatient psychiatric assessment). We also aimed to identify their access and use of technology, along with obstacles to attending clinic appointments, as these factors can influence feasibility of telepsychiatry and its perceived usefulness. This study is informed by the Technology Acceptance Model (TAM) (12), which has been used to assess acceptance towards technology in health care [e.g., (13)]. According to TAM, attitudes towards a technology (i.e., whether it is useful and easy to use) influence the future use of the technology (14). Understanding the acceptability of an innovation can help to inform strategies for improving adoption and actual use.

## Methods

This survey study is the first phase of a larger project evaluating the implementation of telepsychiatry services in an urban setting. The study received approval from the scientific and ethics review board of the University of Montreal Hospital Centre (CHUM) (#17.073), a general hospital located in downtown Montreal, Canada.

### Study Design, Setting and Recruitment

Using a cross-sectional survey design and convenience sampling, participants were recruited from an early intervention program for FEP at CHUM: Clinique JAP—Jeunes Adultes Psychotiques, which also includes a sub-team EQIIP SOL—Équipe d’Intervention Intensive de Proximité, focused on delivering services to youth experiencing concurrent FEP and housing instability. Within this setting, up to 26% of patients with FEP experience homelessness either prior to receiving, or during, specialized services (15). At any given time, the Clinique JAP team provides services to approximately 260–300 youth, and EQIIP SOL has about 30 active patients.

Participants were recruited from the clinic’s waiting room between July 11 and October 31st, 2017. Verbal informed consent was obtained by the research assistant based on the informed consent section at the beginning of the questionnaire; no personal identifiable information was recorded in this study. The anonymous questionnaire was administered by the research assistant in the waiting room or in a quiet room nearby based on participant preference. Respondents were provided with a gift card ($15 CAD) upon completing the questionnaire.

### Data Collection

The paper-based questionnaire had 46 questions with multiple choice, Likert scale, and open-ended options. The questionnaire was adapted from a previously published study, the objectives of which were to better understand access and use of technology and preferences of using technology for a range of mental health services (6, 7). The sample from that study was recruited from a different clinical program for FEP, within the same city. We adapted the previous questionnaire as follows: 1) updated sections on access and use of technology and demographic questions, 2) replaced the rest of the questionnaire with sections on obstacles to attending appointments (e.g., finding time, financial, public transportation, physical limitations, etc.), satisfaction with services, attitudes towards technology, and perspectives on telepsychiatry. Topics were selected based on factors considered to influence perceived usefulness of, and intentions to use technology based on the TAM model. Items were developed through discussion with physician and non-physician clinicians working with this population and members of the research team. Before finalizing the questionnaire, we sought input from additional service providers and young adult patients for comprehensibility and relevance, and pilot tested it with two patients for duration. A copy of the questionnaire items is provided as **Supplementary Material**.

### Data Management and Analysis

The data was entered into a password protected excel file and stored on a secure institutional server. Descriptive statistics were used to summarize the survey responses. Qualitative descriptive analysis was used to synthesize the responses to the open-ended questions pertaining to concerns and recommendations of using videoconferencing/telepsychiatry to communicate with treatment providers. Specifically, all responses were first entered into the excel file, then responses to each question were open-coded (inductively) by two members of the research team, and subsequently all codes were grouped into broader categories, which were discussed and finalized through team discussion. Then, we counted the number of times each of these categories were labelled to identify frequencies (e.g., number of times responses were labelled with the category of ‘confidentiality’).

## Results

### Participants

In total, 83 individuals were approached to participate in the study, from which 51 (61%) consented to participate and completed the questionnaire, which took 16 min on average to complete. The mean age of the participants was 26.1 (sd = 4.2; age range 19–38), of which 59% (n = 30) identified as male; and, 75% (n = 38) had at least a high school diploma. **Table 1** presents the demographic details of the participants. Compared to the clinic’s population, the sociodemographic characteristics of our sample was lower in terms of gender (e.g., 59% vs. 80%) (5), however, it is generally consistent with the broader literature to have more males than females diagnosed with FEP (16). In addition, our sample was generally consistent with the clinic population in terms of having a high school diploma (75% vs. 69%), and in terms of mean age—given that participants could be at any stage of their 5‐year treatment in the program (5).

**Table 1:** Demographic characteristics (n = 51).

Demographic characteristics (n = 51, Mean age = 26.1; Standard deviation = 4,2; Range = 19–38)		
	*N*	%
Sex		
Male	30	59%
Female	20	39%
Other	1	2%
Team		
Clinique JAP	41	80%
EQIIP SOL (youth with housing instability)	10	20%
Level of education^*^		
University, completed or no	18	36%
College, completed or no	9	18%
High school diploma	11	22%
High school, incomplete	11	22%
Elementary school	1	2%
Current situation/main activity		
Student	14	27%
Employed: Full-time	8	16%
Employed: Part-time	7	14%
I do not have a job/I do not go to school	13	25%
Other	6	12%
Volunteer	3	6%
Current living situation (more than one response possible)		
Alone in an autonomous apartment	16	31%
Apartment with roommates	12	24%
Supervised apartment	9	18%
Group home/Youth centre	7	14%
With family	7	14%
Hospitalized	7	14%
On the streets	1	2%
Shelter/dormitory	1	2%

*Data for the Level of education was only reported for 50 participants.

### Access and Use of Technology

As illustrated in **Table 2**, the majority of participants had a smartphone (84%, n = 43) and access to a personal computer in terms of either a laptop (55%, n = 28) or a desktop (33%, n = 17). Many participants also reported access to a public computer (61%, n = 31) or a computer belonging to someone else (37%, n = 19). A smaller percentage reported access to an iPad/tablet (27%, n = 14) and a few reported having cell phones without any Internet connection (14%, n = 7).

**Table 2:** Access to technology among young adults with first-episode psychosis treated in an urban early intervention service (n = 51).

Technology device	Total*	%
Smartphone	43	84%
Public computer	31	61%
Personal laptop computer	28	55%
Laptop or desktop computer belonging to a friend, family member, etc.	19	37%
Personal desktop computer	17	33%
iPad/tablet	14	27%
Cell phone with no Internet connection	7	14%

^*^Multiple responses possible.

The majority had access to a home Internet plan (76%, n = 39) and a little over half had access to a cellular data plan (55%, n = 28). Many reported accessing the Internet in public spaces (69%, n = 35), at school (27%, n = 14), or work (14%, n = 7). Several (43%, n = 22) reported having access to both a home Internet plan and a cellphone plan. In terms of frequency of Internet access, the majority reported daily use (78%; n = 40), the rest reported using it at least once per week (6%, n = 3), once per month (4%, n = 2), irregularly (10%, n = 5), or never (2%, n = 1). The majority of the homeless participants (80%, n = 8/10) reported accessing the Internet through public settings.

**Figure 1:**
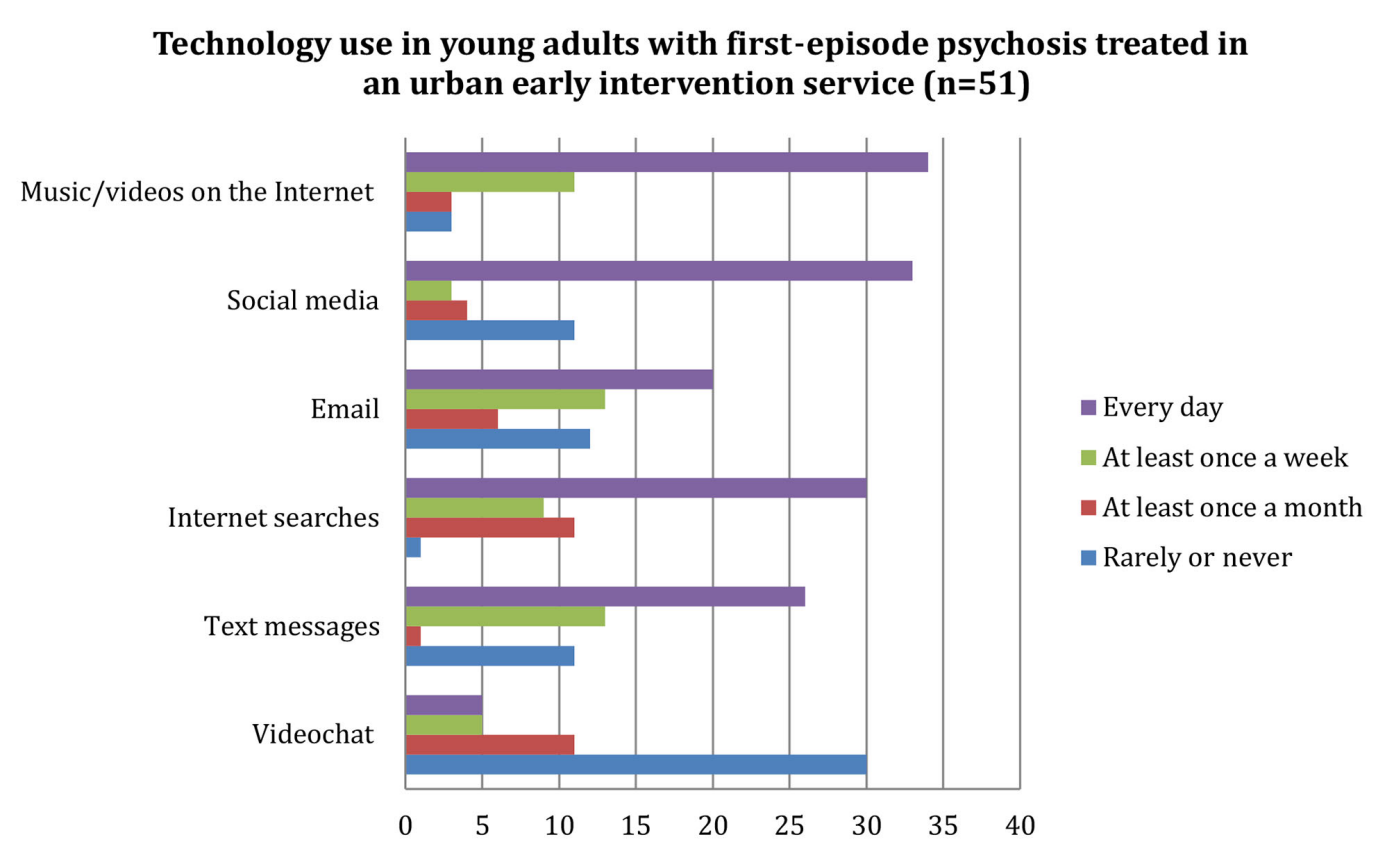
Technology use among young adults with first-episode psychosis treated in an urban early intervention service (n = 51). Horizontal axis represents number of participants.

As illustrated in **Figure 1**, the majority reported listening to music/watching videos (89%, n = 45) and using social media (71%, n = 36) on a daily basis or at least once a week. More than half (59%, n = 30) rarely or never used video chat technologies (e.g., Skype, Google Hangouts, Facetime, others).

When asked about their use of technology to communicate with their treatment team, most reported never using text messaging (94%, n = 48), and the rest reported once a week (4%, n = 2) or once a year use (2%, n = 1). More than half reported never using email to communicate with their treatment team (69%, n = 35), and the rest reported use once a week (10%, n = 5), once a month (12%, n = 6), or once a year use (10%, n = 5). None reported using video chat or social media to communicate with their treatment team.

In terms of competency with video chatting, less than half reported feeling very competent with video chatting (43%, n = 22); the rest reported feeling somewhat competent (27%, n = 14), slightly incompetent (8%, n = 4), incompetent (18%, n = 9), and two did not provide an answer.

### Obstacles to Attending Appointments

As illustrated in **Table 3**, several participants (37%, n = 19) identified two or more obstacles to attending their clinic appointments, many reported at least one obstacle (41%, n = 21), whereas a few (22%, n = 11) did not report any obstacles at all. The most common obstacles were symptom-related difficulties (e.g., anxiety; 43%, n = 22) and scheduling difficulties (e.g., in relation to work or school; 41%, n = 21). Other obstacles were financial difficulties pertaining transportation (20%, n = 10), access to public transportation (20%, n = 10), physical limitations (10%, n = 5), and not knowing how to get to the hospital (8%, n = 4).

**Table 3:** Obstacles to attending clinic appointments (n = 51).

Obstacles to attending clinic appointments	Total^*^	%
Symptoms (e.g., anxiety)	22	43%
Scheduling (e.g., in relation to work, school)	21	41%
No difficulties reported	11	22%
Finances for transportation	10	20%
Access to public transportation	10	20%
Physical limitations	5	10%
Not knowing how to get to the hospital	4	8%
Neutral/I do not wish to answer the question	0	0%

^*^Multiple responses possible.

### Perceptions of Telepsychiatry Services

As illustrated in **Table 4**, in terms of receptivity towards the idea of receiving telepsychiatry services (i.e., communicating with service providers using a secure videoconferencing platform), 49% (n = 25) said they were very favorable and 25% (n = 13) were somewhat favorable towards the idea. The rest indicated that they were somewhat unfavorable (14%, n = 7), very unfavorable (6%, n = 3), did not know (4%, n = 2), or preferred not to answer (2%, n = 1). When asked more directly whether they would like to attend a future clinic appointment *via* a secure videoconferencing platform, 55% (n = 28) indicated to be interested and 27% (n = 14) probably interested, with the rest stating no (18%, n = 9).

**Table 4:** Receptivity towards the idea of receiving telepsychiatry services (n = 51).

Receptivity towards the idea of receiving telepsychiatry services	Total	%
Very favorable	25	49%
Somewhat favorable	13	25%
Somewhat unfavorable	7	14%
Very unfavorable	3	6%
I don’t know	2	4%
I do not wish to answer the question	1	2%

As illustrated in **Table 5**, the majority indicated that telepsychiatry services should be used in unforeseen or emergency situations (75%, n = 38) and as a last resort when face-to-face meetings are not possible (75%, n = 38). Similarly, 65% (n = 33) reported that this approach could be used to avoid traveling to the hospital (61%, n = 31), while 37% (n = 19) indicated that telepsychiatry services could be used to replace in-person meetings.

**Table 5:** Situations in which telepsychiatry could be used (n = 51).

Situations in which telepsychiatry could be used	Total^*^	%
In the case of unexpected events or in an emergency	38	75%
As a last resort when in-person meetings are impossible	38	75%
To avoid travelling to the hospital	31	61%
To replace in-person meetings	19	37%
Other	4	8%

^*^Multiple responses possible.

More than half identified ease of use (57%, n = 29), and confidentiality and security (57%, n = 29) as essential characteristics of a video conferencing platform. Some participants indicated cost (29%, n = 15), efficiency (24%, n = 12), quality of sound (18%, n = 9), and quality of image (14%, n = 7) as being essential.

#### Concerns and Recommendations

Of the 51 participants, 30 provided responses to the open-ended question on whether they had any concerns about the use of videoconferencing/telepsychiatry with their treatment providers. Ten out of these 30 respondents stated that they did not have any concerns regarding telepsychiatry services. For the remainder 20, the most common concerns (i.e., mentioned by at least 4 participants or more) were about “loss of human contact.” For example, one participant stated, “I do not want it to replace meetings in person” (P14), and another expressed concerns that telepsychiatry “will replace face-to-face consultations” (P21). Another common concern was confidentiality. For example, one participant expressed concerns about the “possibility that calls will be recorded” (P46), another stated, “I would not want it to be recorded” (P2), and another asked, “to what extent is it confidential?” (P30). Other concerns were: costs associated with the use of the service; quality of the transmission in terms of image and sound; not having access to the appropriate environment (e.g., sound-proof/noise free environment); not having the competency or skills to use the technology; reliability of video conferencing for making diagnoses; and “being disturbed in private life, feeling harassed” (P50).

Of the 51 participants, 29 participants provided responses to the open-ended question on recommendations. The most common recommendation pertained to ensuring confidentiality. For example, one participant stated, “confidentiality, zero on social media” (P1) and others stated, “ensure confidentiality” (P11) or “maintain confidentiality” (P45). Another common response pertained to ensuring quality of the technology and its transmission, for example to “test it before, be certain that it works well in terms of sound and image” (P2). Participant recommendations also pertained to accessibility; for example: ensuring there is no cost to users, making it available on multiple devices, using simple passwords, and having a color code for the type of calls (e.g., urgent, not urgent). They also highlighted the importance of training and orientation; for example, one participant recommended that there be a “tutorial on the platform” (P41). Others highlighted that it should be used as a complementary method as one participant stated, “it should not be obligatory” (P38) and another stated, “use it as a last resort, like during travel, or studying out of town” (P30).

## Discussion

Previous studies [e.g., (5–7, 17)] have examined access and use of technology in populations diagnosed with FEP, however to our knowledge, this is the first study that has a focus on the use of telepsychiatry to attend outpatient follow-up appointments. Telepsychiatry is a specific type of digital service that requires real-time presence of both parties, and as such differs quite significantly with other types of digital services, such as using websites or social media for information, or completing online psychoeducation modules autonomously. Our study focuses on patient perceptions regarding the use of videoconferencing solutions to attend a clinic appointment and considers how this might help to address barriers to engaging with their follow-up. In addition, 20% of our sample had a recent history of being homeless or were experiencing housing instability at the time of the study. With larger samples, future research could be focussed on more in-depth study of the potential role of telepsychiatry for this hard-to-reach population.

Our main findings were that the majority of the participants experienced obstacles (e.g., finding time, transportation) to attending their follow-up appointments, and more than a third faced multiple obstacles. Most had access to and used mainstream technologies (e.g., Internet, computers, smartphones) but did not use them to communicate with their treatment team. More than half expressed interest in telepsychiatry but had limited experience with video chat technologies. The most common concerns towards telepsychiatry were loss of human contact and confidentiality.

In-depth research is needed to better understand the obstacles young adults with FEP face in attending their follow-up appointments and whether ambivalence towards telepsychiatry is related to factors such as: limited social and productive opportunities to use videoconferencing technologies, cognitive difficulties, or residual symptoms of psychosis (e.g., emotional withdrawal, hallucinations, delusional thinking). Regarding the latter, symptoms could have influenced participant concerns (e.g., being recorded could be a delusional preoccupation); however, whether related to symptomology or not, our findings suggest that privacy and confidentiality issues are important to discuss with patients prior to providing telepsychiatry services.

In addition, the low levels of use and perceived competencies with mainstream video chat technologies in this study indicate the importance for providing training and technical support for patients when implementing telepsychiatry, even if the target population is young. Moreover, this training needs to account for cognitive difficulties and symptomatology found in the FEP population and provide reassurance that telepsychiatry is not meant to replace human contact. This study highlights the importance of integrating telepsychiatry into hybrid models of care, wherein telepsychiatry is offered as part of many contact possibilities, and as a complement to ‘in person’ contact when the latter is not feasible for patients. Furthermore, our findings indicate that ensuring patients have access to the Internet from a location that affords them quality of sound and image and privacy is a critical consideration for providing telepsychiatry services and may be particularly relevant for those living in unstable housing situations.

The limited use of technology in this population to communicate with healthcare providers could also be related to lack of clinician acceptance, which has been shown to be one of the most critical factors in determining telepsychiatry implementation (18). Research has shown that clinicians fear that videoconferencing could make contact “less personal” and challenging to establish trust (19). It is also possible that clinicians may lack the confidence and skills to use these technologies with patients. Clinician acceptance can be influenced by access to training, practice guidelines on the use of telepsychiatry in early intervention programs for psychosis, the latest technologies, and institutional support, all of which are key factors to consider for future research, policy, and practice.

### Limitations

There are certain limitations of this study. The small sample size limits the representativeness of our sample and could have resulted in missing some perspectives. In addition, we used a non-validated questionnaire, which poses limitations on the consistency and accuracy of our results. Moreover, our instrument included mostly closed-ended questions, thus, we did not have access to detailed data on participant perspectives. Also, approximately 40% of the participants did not answer the open-ended questions which were at the end of the questionnaire, possibly due to fatigue. Qualitative research is needed to better understand the views of young adults regarding urban telepsychiatry, for example through interviews and focus groups. In addition, our recruitment occurred during summer and early fall months, which is relevant for a northern community where winter weather can influence patient perceptions of obstacles to attending appointments and receptivity towards telepsychiatry. For example, colder weather and snowy/icy conditions on the road can reduce motivation to leave the house and can also have an impact on the accessibility of public transportation. Furthermore, recruitment was done in the waiting room, among patients attending their appointments. The results could have been different if we surveyed individuals disengaging from care. For example, it is possible that receptivity to telepsychiatry could be different among individuals who wish to attend appointments but do not attend because of other commitments (such as school or work) versus those who are reluctant to engage with care due to other factors such as stigma. Research with large and representative samples is warranted to assess how these factors may influence acceptability towards telepsychiatry.

## Conclusion

This study addresses an important gap in the literature on the use of telepsychiatry services for outpatient follow-up, from the perspectives of patients receiving treatment for FEP in an urban setting. While several participants faced multiple obstacles to attending their appointments and were receptive towards telepsychiatry, they did not have access to this service. It is important to provide education to clinicians working in urban settings on the potential for telepsychiatry in helping overcome obstacles to attending clinic appointments. Moreover, concerns regarding loss of human contact, confidentiality and privacy, costs, quality of sound and image, reliability for assessing, and perceived competencies in using videoconferencing technologies need to be addressed prior to offering telepsychiatry services. Finally, in the context of large scale, global implementation of early intervention for psychosis services, where distance, poor public transport, stigma, and competing priorities are an issue in accessing care, telepsychiatry can be a relevant add-on to current services and warrants further attention at the levels of research, policy, and practice.

## Data Availability Statement

The datasets for this article are not publicly available because participants recruited in this study did not give their consent to their raw data being publicly shared even if anonymised. Additional information regarding the datasets should be directed to Shalini Lal, shalini.lal@umontreal.ca.

## Ethics Statement

This study was carried out in accordance with the recommendations of the scientific and ethics review board of the University of Montreal Hospital Centre (CHUM) (#17.073) with informed consent from all subjects. 

## Author Contributions

SL and AA-B contributed to the conception and design of the study. SL wrote the first draft of the study protocol and survey. AA-B, SS, FB, and IS contributed to revisions of the protocol, including survey development. SL led the study implementation, analysis, and reporting. FB contributed to recruitment and data collection. JW conducted data entry; JW and SS verified, analyzed, and reported the results. SL wrote the first draft of the manuscript. AA-B and SS contributed to revising the second draft. All authors reviewed and approved the final manuscript.

## Funding

This research was partially funded by a Junior Research Scholar establishment grant from the Fonds de recherche du Québec—Santé (FRQS) awarded to SL and an undergraduate scholarship award through the “PRogramme d’Excellence en Médecine pour l’Initiation En Recherche” (PREMIER) awarded to FB. SL is supported by the Canada Research Chairs Program and was previously supported by a New Investigator Salary Award from the Canadian Institutes of Health Research and a Junior Research Scholar Salary Award from the Fonds de recherche du Québec—Santé (FRQS).

## Conflict of Interest

The authors declare that the research was conducted in the absence of any commercial or financial relationships that could be construed as a potential conflict of interest.
