# Supplementary material for: Perspectives of Young Adults on Receiving Telepsychiatry Services in an Urban Early Intervention Program for First-Episode Psychosis: A Cross-Sectional, Descriptive Survey Study
Source: Front Psychiatry. 2020 Mar 3;11:117. doi: 10.3389/fpsyt.2020.00117 (PMC7065530; doi:10.3389/fpsyt.2020.00117)
Supplement: Supplementary file 1 [file DataSheet_1.pdf]

Supplementary Material

- *Questionnaire*

ID#

1. Which technological devices do you have access to? (check all that apply)

1☐Smartphone

2☐Cell phone with no Internet connection

3☐Ipad/Tablet

4☐Personal laptop computer

5☐Personal desktop computer

6☐Laptop or desktop computer belonging to a friend, family member, etc.

7☐Public computer (e.g., library, community center, etc.)

2. What are the obstacles that prevent you from attending JAP/SOL clinic appointments? (check all that apply)

1☐I have **physical** difficulties in getting around

2☐I have **financial** difficulties in getting around

3☐I have difficulties **going outside (anxiety, not wanting to go outside)**

4☐I have difficulties **finding time** (busy schedule, studies, work, etc.)

5☐I have difficulties **using public transportation** to get around (too far, not enough buses or metros)

6☐I have difficulties **finding my way around** (I don't know how to get to the hospital)

7☐**I don't have any particular difficulties**

-2☐Neutral/I do not wish to answer the question

3. How often do you access the Internet? (only one answer)

1☐Never

2☐Irregular access

3☐Once per month

4☐At least once a week

5☐Every day

4. How do you access the Internet? (check all that apply)

1☐Cell phone plan

2☐Home Internet plan

3☐At school or in an educational institution

4☐At work

5☐In cafés, public spaces (e.g., library)

5. Generally, <u>to what extent</u> are you SATISFIED with the outpatient SERVICES RECEIVED from the JAP/SOL team? (only one answer)

1☐Very unsatisfied

2☐Somewhat unsatisfied

3□Moderately satisfied
4□Very satisfied
5□I do not know
-2□I do not wish to answer the question

6. Generally, to what extent do you consider the OUTPATIENT SERVICES TO BE FLEXIBLE (i.e. adapt to your schedule, adjust to your needs, etc.)? (only one answer)

1□Not at all flexible
2□Somewhat flexible
3□Moderately flexible
4□Very flexible
5□I do not know
-2□I do not wish to answer the question

What method of communication have you used to KEEP IN TOUCH WITH YOUR JAP/SOL clinician during the past year? (only one answer for each question)

	Every day 5□	At least once a week 4□	At least once a month 3□	At least once a year 2□	Never 1□
**7. In person (at the clinic)**	○	○	○	○	○
**8. In person (in the community)**	○	○	○	○	○
**9. Through my parents**	○	○	○	○	○
**10. Phone**	○	○	○	○	○
**11. Text messages (SMS)**	○	○	○	○	○
**12. Social media (e.g., Facebook, Snapchat)**	○	○	○	○	○
**13. Email**	○	○	○	○	○
**14. Videochat (i.e. Skype, Google hangouts, Facetime, others)**	○	○	○	○	○
**15. Other (please specify) ……**	○	○	○	○	○

16. Generally, to what extent are you <u>satisfied</u> with the METHODS OF COMMUNICATION that you use with the JAP/SOL clinicians?
1= absolutely not satisfied and 10 = absolutely satisfied

1 Absolutely not satisfied	2	3	4	5	6	7	8	9	10 Absolutely satisfied	-1☐ I do not know	-2☐ I do not wish to answer the question

17. Generally, to what extent are you <u>satisfied</u> with the face-to-face CONSULTATIONS with JAP/SOL clinicians?
1= absolutely not satisfied and 10 = absolutely satisfied

1 Absolutely not satisfied	2	3	4	5	6	7	8	9	10 Absolutely satisfied	-1☐ I do not know	-2☐ I do not wish to answer the question

For questions 18 to 22, please circle the answer that best reflects your opinion on technology (Internet, cell phones, tablets, etc.) (only one answer for each question)

	1☐ **Completely disagree**	2☐ **Partially disagree**	3☐ **I do not wish to answer the question**	4☐ **Partially agree**	5☐ **Completely agree**
**18.** Technology plays a central role in daily life					
**19.** Technology enables me to connect with others, the outside world, and to stay informed					
**20.** Technology represents positive progress					
**21.** Technology helps to be integrated with society					
**22.** Technology helps with finding support, assistance, and information					

<u>Indicate the FREQUENCY with which you use</u> each technology listed below (only one answer).

	**23.** Videochat (e.g., Skype, Google Hangouts, Facetime, others)	**24.** Text messages	**25.** Internet searches	**26.** Email	**27.** Social media	**28.** Music/videos on the Internet
1☐ Rarely or Never						
2☐ At least once a month						
3☐ At least once a week						
4☐ Every day						

For each technology listed below, evaluate <u>YOUR LEVEL OF COMPETENCY</u> (only one answer for each question).

	**29.** Videochat (Skype, Google Hangouts, Facetime, others)	**30.** Text messages	**31.** Internet searches	**32.** Emails	**33.** Social media	**34.** Music /videos on the Internet
1☐ Incompetent						
2☐ Slightly incompetent						
3☐ Somewhat competent						
4☐ Very competent						
-2☐ I do not wish to answer the question						

35. To what extent would you be favourable towards the idea of communicating using videoconferencing technology with JAP/SOL clinicians as part of your care? (only one answer)

1☐ Very unfavourable
2☐ Somewhat unfavourable
3☐ Somewhat favourable
4☐ Very favourable
5☐ I don't know
-2☐ I do not wish to answer the question

36. What should be the 2 ESSENTIAL CHARACTERISTICS of a videoconferencing platform? (maximum 2 answers)

1☐ Easy to use
2☐ Good sound quality
3☐ Confidential and secure
4☐ Efficient
5☐ Good image quality
6☐ Cost
7☐ Other: ________________

37. In your opinion, a videoconferencing tool should be used to (check all that apply):

1☐ To replace in-person meetings
2☐ As a last resort when in-person meetings are impossible
3☐ In the case of unexpected events or an in an emergency
4☐ To avoid having to travel to the hospital
5☐ To facilitate/increase access to care
6☐ Other: __________________________

38. WOULD YOU LIKE TO TRY VIDEOCONFERENCING in a future consultation?

1☐ No
2☐ Yes
3☐ Maybe

39. Do you have CONCERNS about the use of VIDEOCONFERENCING with the JAP/SOL team? If yes, please specify:

____
____
____
____

40. Do you have RECOMMENDATIONS regarding the use of VIDEOCONFERENCING with the JAP/SOL team? If yes, please specify:

____

____

____

____

THE INFORMATION IN THE NEXT SECTION WILL NOT BE USED TO IDENTIFY THE PERSON FILLING OUT THIS SURVEY

41. Age: ____

42. Are you:

1□ A man

2□ A woman

43. Which of the following options best describes your current living situation? (check all that apply)

1□ On the streets

2□ Shelter / dormitory

3□ Group home / Youth centre

4□ With friends

5□ With family

6□ Supervised apartment

7□ Alone in an autonomous apartment

8□ Apartment with roommates

9□ I occupy uninhabited or abandoned places

44. Level of education completed

1□ Elementary school

2□ High school, incomplete

3□ High school diploma

4□ CEGEP, completed or no

5□ University, completed or no

45. Which of the following categories best describes your current situation/main activity? (only one answer)

1□ I do not have a job / I do not go to school

2□ I have a job

Id work: 2□ Full-time 3□ Part-time

4□ Student

5□ Volunteer

6□ Other (please specify) ………………

46. Are you followed by: 1□ JAP 2□ SOL

46.1 Since when? Indicate the year and month of your first admission:

……………………………………………………………………………………

We thank you for your participation.

Interviewer notes

____

____

____

____

____

____

Interviewer initials: _____
